# The Small Rho GTPase TC10 Modulates B Cell Immune Responses

**DOI:** 10.4049/jimmunol.1602167

**Published:** 2017-07-26

**Authors:** Marianne Burbage, Selina J. Keppler, Beatriz Montaner, Pieta K. Mattila, Facundo D. Batista

**Affiliations:** * Lymphocyte Interaction Laboratory, Francis Crick Institute, London NW1 1AT, United Kingdom;; † Institute of Biomedicine, Unit of Pathology and MediCity Research Laboratories, University of Turku, BioCity, 20520 Turku, Finland; and; ‡ Ragon Institute of MGH, MIT and Harvard, Cambridge, MA 02139

## Abstract

Rho family GTPases regulate diverse cellular events, such as cell motility, polarity, and vesicle traffic. Although a wealth of data exists on the canonical Rho GTPases RhoA, Rac1, and Cdc42, several other family members remain poorly studied. In B cells, we recently demonstrated a critical role for Cdc42 in plasma cell differentiation. In this study, we focus on a close homolog of Cdc42, TC10 (also known as RhoQ), and investigate its physiological role in B cells. By generating a TC10-deficient mouse model, we show that despite reduced total B cell numbers, B cell development in these mice occurs normally through distinct developmental stages. Upon immunization, IgM levels were reduced and, upon viral infection, germinal center responses were defective in TC10-deficient mice. BCR signaling was mildly affected, whereas cell migration remained normal in TC10-deficient B cells. Furthermore, by generating a TC10/Cdc42 double knockout mouse model, we found that TC10 can compensate for the lack of Cdc42 in TLR-induced cell activation and proliferation, so the two proteins play partly redundant roles. Taken together, by combining in vivo and in vitro analysis using TC10-deficient mice, we define the poorly studied Rho GTPase TC10 as an immunomodulatory molecule playing a role in physiological B cell responses.

## Introduction

B cells constitute an essential part of the adaptive immune system by producing Abs that target pathogens and infected cells for destruction. This humoral immune response is driven by highly specific interactions between different Ags and the BCR. Ag binding to BCR triggers intracellular signaling, leading to cell proliferation and differentiation into Ab-producing plasma cells. Multiple studies in the last decade have established that the Ags recognized by B cells in vivo are predominantly bound to the surface of APC (1, 2). This indicates that Ag recognition mostly occurs within the immunological synapse, a specialized and highly dynamic cell-cell interaction structure that functions as a platform for receptor signaling and signal regulation as well as for Ag gathering and internalization (3). The formation of the immunological synapse requires substantial morphological changes in B cells (4) that heavily depend on the actin cytoskeleton (5–7). In contrast, recent studies indicate an important role for actin in the regulation of BCR signaling by controlling the dynamics and organization of the BCR and its coreceptors within the plasma membrane (8–12). These specific features, combined with the well-known roles of the cytoskeleton in cell migration, cell division, and intracellular trafficking, have placed the actin cytoskeleton as a major player in B cell activation.

The actin cytoskeleton is a highly dynamic cellular network that provides localized force and stability to various cellular structures. Actin filaments (F-actin) are constantly formed, disassembled, or cross-linked according to the changing needs of the cell. The spectrum of different cellular functions controlled by the actin cytoskeleton necessitates a highly sophisticated and adaptable network of actin regulatory proteins that orchestrate different steps of the actin dynamics in a highly spatio-temporally controlled manner downstream of various cellular cues. A major role in signaling to different actin-binding proteins is played by the Rho family of small GTPases (Rho GTPases) that cycle between the inactive GDP-bound and active GTP-bound states (13). The importance of Rho GTPases in B cell activation is illustrated, for example, by the defects in humoral immune responses linked with mutations in Rho GTPase activating guanosine nucleotide exchange factors, Vav and DOCK8 (14–17). The most studied Rho GTPases are RhoA, Rac, and Cdc42, which are highly conserved from yeast to mammals. RhoA, Rac, and Cdc42 control a multitude of actin-regulatory pathways to categorically induce actin stress fibers, lamellipodia, or filopodia, respectively. In B cells, important functions have been identified for all these three Rho GTPases. RhoA is required for inositol trisphosphate and calcium signaling, critical pathways downstream of the BCR (18). Rac1 and Rac2 can to some extent compensate each other, but the lack of both these family members leads to a block in B cell maturation (19, 20). Recently, we found dramatically defective Ab responses in a conditional mouse model bearing a B cell–specific deletion of Cdc42. This striking phenotype was caused by a failure of Cdc42-deficient B cells to differentiate into plasma cells (21, 22). Cdc42-deficient B cells also showed reduced BCR signaling and presentation of internalized Ag. However, Cdc42 has well-established roles in various cellular processes involving the actin cytoskeleton, such as cell migration and endocytosis, which were not detectably altered in Cdc42-deficient B cells, suggesting partial redundancy with other Rho family members.

In total, 23 proteins belong to the family of Rho GTPases. Highest homology to Cdc42 can be found in proteins TC10 (also known as RhoQ) and TCL (also known as RhoJ), which together with Cdc42 form a distinct subfamily of Rho GTPases. TC10 has been mostly studied in adipose and neuronal cells, where its reported functions include insulin-regulated translocation of glucose transporter 4 (GLUT4) (23) and neurite growth (24, 25), respectively. The function of TC10 in both GLUT4 translocation and axonal growth has been pinned to the regulation of exocytosis (26, 27). Also, a role for TC10 in the regulation of actin-based structures like filopodia, stress fibers, and actin cortex has been demonstrated (28, 29). TCL regulates endothelial cell migration, actomyosin contractility, focal adhesions, and angiogenesis (30, 31). Nothing is known about the function of these proteins in B cells. The expression pattern of TCL is endothelial cell–restricted (30, 32) and our protein expression database searches (http://Immgen.org) did not suggest significant expression in lymphocytes. However, the database suggests B cell–specific and relatively high expression levels for TC10. Based on its expression pattern and high homology to Cdc42, TC10 could potentially synergize with Cdc42 in B cell activation. However, the relationship of these two homologous proteins is complex, as both synergistic (25, 33, 34) and antagonistic molecular functions have been indicated (35).

Inspired by the critical functions of Cdc42, we sought to characterize the role of TC10 in B cells and humoral immune response by generating a TC10-deficient mouse. Although B cell–development was not altered, we detected reduced peripheral B cell numbers in this mouse model. The TC10-deficient B cells showed slightly decreased BCR activation but normal Ag internalization and proliferation. Upon immunization, TC10-deficient mice showed defective IgM responses. Also, upon viral infection, we observed a reduced formation of germinal center B cells. By the generation of a TC10/Cdc42 double knockout (KO) mouse strain, we revealed that these proteins play a synergistic role in proliferative responses to TLR agonists. To our knowledge, this study provides the first characterization of the role of TC10 in B cells and adds to the understanding of the immunological functions of the relatively scarcely studied Rho GTPases outside of the archetypes Rac, RhoA, and Cdc42.

## Materials and Methods

### Animal breeding and generation

Animals carrying a conditionally targetable deletion of TC10 were designed in collaboration with Gene Bridges (Heidelberg, Germany) and generated at the transgenic facility of the former London Research Institute, Cancer Research U.K., currently the Genetic Manipulation Service, the Francis Crick Institute (London, U.K.). Cdc42^F/F^ mice were kindly provided by Prof. Brakebusch (36), and mb1-Cre mice, to obtain B cell–specific deletion of Cdc42, were kindly provided by Prof. M. Reth (37). TC10 KO animals were generated by microinjection into C57BL/6J embryonic stem cells of a construct encoding for the exon2 of TC10 flanked by two LoxP sites and a neomycin cassette flanked by two FRT sites and two homology regions allowing insertion into genomic DNA by homologous recombination. Euploid neomycin-resistant clones were injected into BALB/c host blastocysts and transferred to fertilized females. Offspring were screened by fur color, and black pups were genotyped for neomycin and crossed to mice expressing Cre recombinase under the PGK promoter (38), causing deletion at early stages of embryonic development. Genotyping was performed via Transnetyx, using probes targeting RhoQ wild-type (WT) and KO alleles, Cdc42 Flox, or WT alleles, and mb1-cre. Mice were used between 8 and 12 wk of age. Wherever possible, WT littermates were used as controls. Otherwise, age-matched C57BL/6J animals were used. PGK-Cre females as well as μMT (39) and OTII [(40), used for purification of OVA-restricted CD4 T cells] mice on the C57BL/6J background were obtained from the internal facility at the Francis Crick Institute. C57BL/6J mice were purchased from Charles River.

For the generation of mixed bone marrow chimeras, 6–8 wk-old μMT mice were provided acidified water 1 wk before the beginning of the procedure, lethally irradiated using 2 × 6 Gy, and intravenously injected 24 h later with 2 × 10^6^ donor bone marrow cells in adequate proportions. Animals were bled 8–10 wk after adoptive transfer to check the reconstitution efficiency, and were subsequently used for experiments.

Mice were bred and maintained at the animal facility of the Francis Crick Institute. The original TC10 line with conditional potential was cryopreserved (embryos and sperm). All experiments were approved by the Animal Ethics Committee of the Francis Crick Institute and the United Kingdom Home Office.

### Primary cell isolation, labeling, and culture

Splenic naive B or CD4 T lymphocytes were purified using a negative B cell or CD4 T cell purification kit (Miltenyi Biotec) yielding enriched populations of ∼95–98% (B cells) and ∼80% (T cells), respectively. Purified B or T cells were labeled in PBS with 2 μM cell trace violet (CTV) (Invitrogen), 1 μM CFSE (Invitrogen), or 2 μM SNARF-1 (Invitrogen) for 5 min at 37°C, or with 3 μM Indo-1 AM (Molecular Probes) in RPMI 1640 for 30 min. Cells were maintained in complete B cell medium [RPMI 1640 supplemented with 10% FCS, 25 mM Hepes, Glutamax, penicillin streptomycin (Invitrogen), and 1% β-mercaptoethanol (Sigma-Aldrich)].

### Cell culture and transfections

A20 lymphoma cells expressing a hen egg lysozyme–specific IgM BCR with the specificity of D1.3 (41) were maintained in complete B cell medium (described above). Nucleofections were performed on 5 × 10^6^ cells using the Amaxa technology (Kit V, Program L-013), and cells were used 5–6 h after transfection for imaging. Staining with Lysotracker-Green DND26 (Invitrogen) was performed on transfected cells prior to imaging according to the manufacturer’s recommendations.

### Flow cytometry

Single-cell suspensions were prepared from spleen, lymph nodes (LNs), bone marrow, or peritoneal lavage. After blocking the Fc receptors using anti CD16/32 Abs, cells were stained with the appropriate combination of the following Abs: B220 (RA3-6B2), TCRb (H57-597), CD21 (7G6), CD4 (GK1.5), CD23 (B3B4), CD24 (M1/69), CD43 (S7), Ly-51 (6C3), B and T cell activation Ag (GL7), CD95 (Jo2), CD138 (281.2), IgG1 (A85.1), CD5 (53-7.3), CD11b (M1/70), CD19 (1D3), IgM (1B4B1 or II/41), and IgD (11–26). For in vivo labeling of bone marrow progenitors, 2 μg of anti-CD45.2PE Ab were injected i.v., and animals sacrificed precisely 2 min after injection. Bone marrow was subsequently processed and stained with surface markers as specified. Data were acquired on an LSR Fortessa (BD Biosciences) and analyzed with FlowJo (Tree Star).

### Ratiometric Ca^2+^ measurements

Indo-1 AM–labeled naive B cells were analyzed by flow cytometry. Baseline fluorescence was recorded for 25 s, after which 5 μg ml^−1^ anti-κ (HB-58; American Type Culture Collection) or 1 μM Latrunculin A (Calbiochem) were added to the cells, and fluorescence recorded for 5 min on an LSR Fortessa (Becton Dickinson). The fluorescent ratio (405/525) was determined using FlowJo (Tree Star).

### Immunoblotting

Purified B cells were left at 37°C for 15 min in chamber buffer (PBS, 0.5% FCS, 1 g l^−1^ D-glucose, 2 mM MgCl_2_, 0.5 mM CaCl_2_) to equilibrate prior to stimulation. They were then stimulated for various times with 10 μg ml anti-IgM F(ab′)2 fragment (Jackson ImmunoResearch). Stimulated cells were lysed in lysis buffer [20 mM Tris-HCl (pH 8), 150 mM NaCl, 5 mM EDTA, Protease Inhibitor mixture (Roche), 10 mM NaF, 1 mM Na_3_VO_4_, 1% NP40] for 30 min on ice and samples loaded into Tris Glycin gels prior to electrophoresis using the miniprotean system (Bio-Rad). Proteins were detected with Abs against pErk, pAkt (S473), pCD19, pSyk, pSrc, total Erk (all from Cell Signaling Technology), tubulin (Sigma-Aldrich), or TC10 (Proteintech) using the secondary HRP-conjugated anti-rabbit or anti-mouse Abs (Jackson ImmunoResearch). Blot densitometry analysis was performed using Image J software.

### Southern blotting

Genomic DNA was extracted from mouse ear samples in DNA lysis buffer [100 mM Tris (pH 8), 5 mM EDTA (pH 8), 0.2% SDS, 200 mM NaCl] complemented with proteinase K at 56°C from 1 to 2 h. DNA purity was improved using phenol-chloroform extraction, and DNA precipitated using isopropanol. Then 30 μg DNA was digested with adequate restriction enzymes in the presence of spermidine, and separated on a 0.8% agarose gel by electrophoresis at 30 V. Gels were denatured for 30 min in denaturation buffer (1.5 M NaCl, 0.5M NaOH) and neutralized for 45 min in neutralization buffer [1 M Tris-HCl (pH 7.4), 1.5 M NaCl]. Separated DNA was transferred onto a membrane using capillary transfer in 10× SSC buffer overnight. Probes were prepared using the digoxigenin-11-dUTP (DIG)-labeling system (Roche) following the manufacturer’s instructions. Briefly, the target sequence was amplified from genomic DNA by PCR using appropriate primers in the presence of DIG-labeled nucleotides. Hybridization and detection were performed using the DIG detection (Roche) according to the manufacturer’s instructions.

### Chemotaxis assays

Briefly, 6 × 10^5^ naive or preactivated B cells in migration buffer (RPMI 1640, 0.5% BSA, 5 mM Hepes) were placed in the top part of a Boyden chamber containing 5 μm^2^ pores (Corning Costar). CXCL12, CXCL13, CCL19, or CCL21 (R&D Systems) were placed in the bottom part of the chamber at various concentrations.

### Microsphere preparation

Streptavidin-coated microspheres, 0.11 μm in diameter (Bangs Laboratories), were incubated with a saturating amount of biotinylated anti-IgM (Southern Biotech) and OVA (Calbiochem). Limiting stimulatory conditions were obtained by increasing the amounts of Ova used for coating while IgM amounts were kept constant. Efficient titration of the IgM signal was measured by flow cytometry.

### Proliferation analysis

CFSE or CTV-labeled cells at a concentration of 10^6^ cells/ml were stimulated in complete B cell medium supplemented with combinations of 0.3–10 μg ml^−1^ of LPS (Sigma) or CpG (Sigma), 1 μg ml^−1^ CD40L (R&D Systems), 10 ng ml^−1^ of IL4 (R&D Systems), or 10 ng ml^−1^ of IL5 (R&D Systems). CFSE or CTV dilution was measured after 4 d by flow cytometry. For B-T coculture, labeled B cells were incubated with coated microspheres for 30 min at 37°C in complete medium, washed with complete medium to remove excess microspheres, and subsequently cultured with labeled OTII T cells in a 1:1 ratio.

### Ag internalization assays

For internalization assays, purified B cells were loaded with soluble biotinylated anti-IgM (Southern Biotech) on ice for 30 min. Cells were then washed with PBS, 2% FCS to remove excess Ag and incubated for 0, 15, and 25 min at 37°C. After fixation with 2% paraformaldehyde, noninternalized anti-IgM was detected with eFluor450 streptavidin (eBioscience).

### Optical microscopy

Planar lipid bilayers containing anti-mouse κ L chain (HB-58; American Type Culture Collection) were prepared in FCS2 chambers (Bioptechs) by liposome spreading as previously described (42). Briefly, Alexa-633 streptavidin (Molecular Probes) was incorporated into lipid bilayers at a density of 30 molecules/μm^2^, to which mono-biotinylated Ag and GPI-linked ICAM [prepared as described previously (42)] was tethered. Assays were performed in chamber buffer at 37°C and imaged with total internal reflection fluorescence (TIRF) microscopy. TIRF images were acquired with an EMCCD camera (iXon3 897; Andor) coupled to a TIRF microscopy system (Cell R; Olympus) with 488, 561, and 640-nm lasers (Olympus). Images were recorded with Cell R software (Olympus) and analyzed with ImageJ software (National Institutes of Health).

Confocal imaging was performed with a Zeiss LSM 780 microscope. The contact of B cells with the planar lipid bilayer was visualized by interference-reflection microscopy using the 640-nm laser. Images were analyzed in Imaris (Bitplane) and ImageJ (National Institutes of Health).

For multiphoton microscopy, explanted popliteal and inguinal LNs were prepared as described (43), and imaged with an upright multiphoton microscope (Olympus), a 25×, NA 1.05 water immersion objective, and a pulsed Ti:sapphire laser (MaiTai HP DeepSee; Spectra Physics) tuned to 820 nm. Emission wavelengths were detected through band-pass filters of 515–560 nm (CFSE) and 590–650 nm (SNARF-1). Multidimensional movies were analyzed with Imaris. Fluorescence bleed-through into the channel with longer wavelength was removed by subtracting the channel with the shorter wavelength.

### Immunization, infection, and ELISA

For infection, animals were infected with 10^4^ PFU Vaccinia virus (VACV; Western Reserve) intra footpad or with 2 × 10^2^ PFU Influenza A virus (PR8 strain) intranasally. Popliteal (for VACV) or mediastinal (for Influenza A) LNs were analyzed by flow cytometry at day 7. For immunization, mice were injected i.p. with 50 μg NP19-keyhole lympet hemocyanin (KLH) (Biosearch Technologies) in 4 mg Alum (Thermo Scientific). Blood samples were taken from the lateral tail vein on day 0, 7, 14, 21, and 28 after immunization. NP-specific Ab titers were detected by ELISA, using NP23-BSA or NP3-BSA for capture, and biotinylated anti-mouse IgM, IgG, IgG2b, IgG2c (all from Southern Biotech), and IgG3 (BD Biosciences). Titers were determined from the dilution curve in the linear range of absorbance. Total Ab levels in serum prior to immunization were measured by sandwich ELISA. For capture, anti-IgM II/41, anti-IgG2b (BD Biosciences), anti IgG2c, (BD Biosciences), anti–IL-6 (clone MP5-20F3; BioLegend) were used. Purified IgM (BD Biosciences), IgG2b (Southern Biotech), IgG2c (Bethyl), or recombinant IL-6 (BioLegend) were used as standards. Biotinylated anti-IgM (553406; BD Biosciences), anti-IgG2b (Southern Biotech), IgG2c (Southern Biotech), or anti-IL6 (Clone MP5-32C11; BD Biosciences) Abs were used for detection. All noncommercial ELISA plates were developed with alkaline-phosphatase streptavidin and phosphorylated nitrophenyl phosphate (Sigma). Absorbance at 405 nm was determined with a SPECTRAmax190 plate reader (Molecular Devices).

### Experimental data and statistical analysis

Sample sizes were chosen based on published work in which similar phenotypical characterization and defects were reported. When applicable, the data for each study group were compared with Student *t* test and *p* values were calculated. Normal distribution of samples was assumed based on published studies with analysis similar to ours.

## Results

**FIGURE 1.**
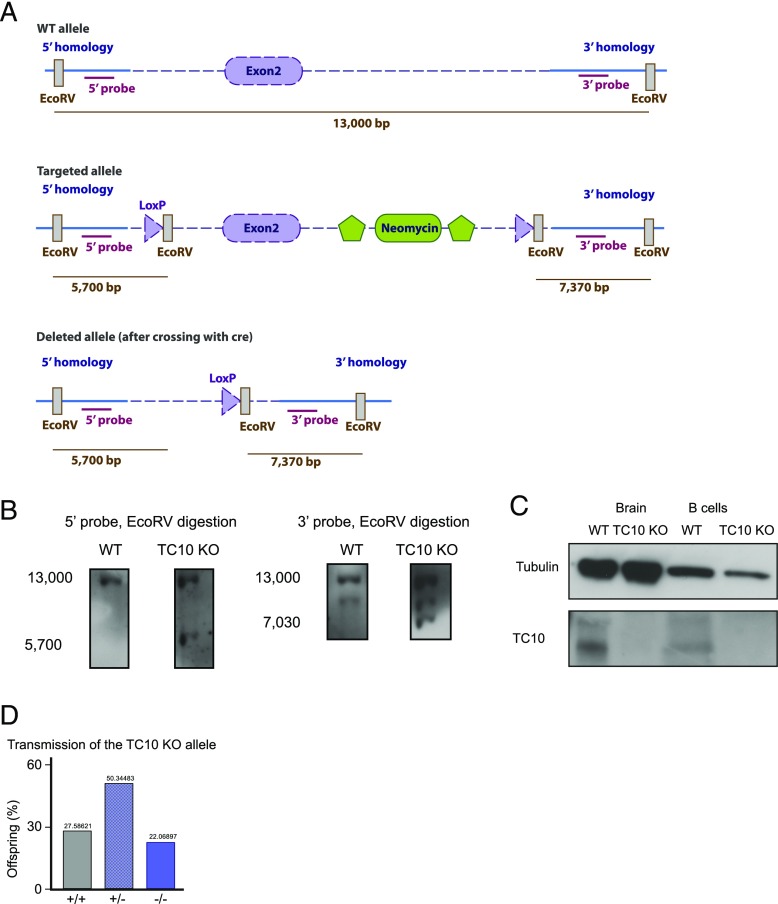
Generation of TC10 KO animals. (**A**) Targeting strategy used for genetic targeting of TC10. The second exon of TC10 is flanked by LoxP sites. A neomycin resistance cassette flanked by FRT sites was used for selection. Crossing these mice to PGK-Cre mice caused excision of the second exon of TC10 in all tissues. (**B**) DNA was purified from the ears of WT and TC10 KO mice (originating from a euploid embryonic stem cell clone, 3A8, injected into blastocysts), digested with *Eco*RV, and submitted to Southern blot analysis. Two probes, depicted in (A), were used for detection, targeting either the 5′ or 3′ homology region. (**C**) Expression of TC10 (lower panel) and tubulin (upper panel) were detected by Western blot in total protein extract from brain or purified B cells of WT and TC10 KO animals. (**D**) Transmission frequencies (calculated over 100–200 pups from heterozygous breedings) of the TC10 KO allele.

To reveal the role of TC10 in B cell function and the humoral immune response, we generated a TC10-deficient mouse model. We targeted the second exon of the TC10 gene by flanking it with LoxP sites. The selection of targeted embryonic stem cells was facilitated by a neomycin cassette and correct insertion into the genome was verified by Southern blot from mice generated from two independent embryonic stem cell clones (Fig. 1A, 1B). The neomycin cassette, together with the second exon of TC10, was cleaved off by crossing the mice to PGK-Cre females, wherein Cre recombinase is expressed in the oocyte (38), generating mice that lack the exon 2 of TC10 in all tissues. Homozygous mice were viable and fertile. Indeed, we observed that TC10 was clearly expressed both in the brain and in purified B lymphocytes in WT animals, but the expression was completely abrogated in animals with deletion in exon 2 of TC10 (Fig. 1C, Supplemental Fig. 1A). These animals developed comparably to their WT littermates, and transmission of the TC10 deletion occurred in agreement with Mendelian prediction (Fig. 1D, Supplemental Fig. 1B). Although there were no apparent phenotypic differences between the strains derived from the two successfully targeted embryonic stem cell clones (data not shown), we chose the mouse strain derived from the clone 3A8 for further experiments and refer to it as TC10 KO.

**FIGURE 2.**
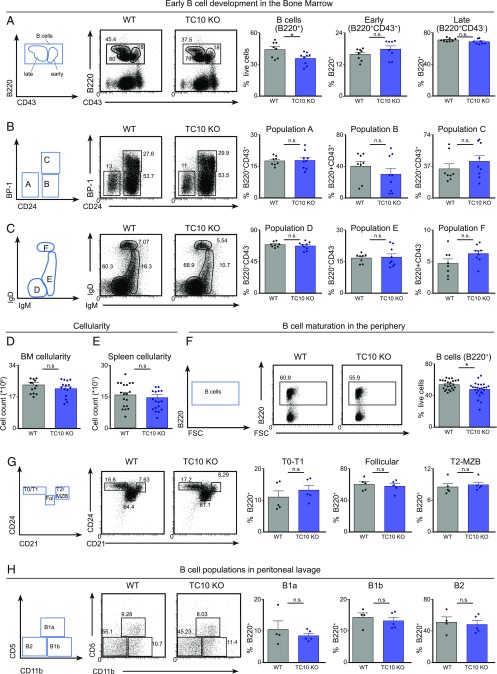
TC10 KO mice show reduction in total B cell numbers but normal developmental subpopulations. Flow cytometry analysis of the bone marrow (**A**–**C**), spleen (**D** and **E**), or peritoneal lavage (**F**) from WT and TC10 KO mice using the gating strategies shown on the left. (A) B cells (B220^+^) were subdivided into early (CD43^+^) and late (CD43^−^) progenitors. (B) Early progenitors were subdivided based on CD24 and BP-1 expression levels into Hardy populations A (B220^+^CD43^+^CD24^−^BP-1^−^), B (B220^+^CD43^+^CD24^+^BP-1^−^), and C (B220^+^CD43^+^CD24^+^BP-1^+^). (C) Late progenitors were separated according to IgM and IgD receptor expression into Hardy populations D (B220^+^CD43^−^IgM^−^IgD^−^), E (B220^+^CD43^−^IgM^+^IgD^−/Int^), and F (B220^+^CD43^−^IgM^+/Int^IgD^+^). Quantifications are shown on the right and represent percentage of live cells (A), of B220^+^CD43^+^ (B), or of B220^+^CD43^−^ (C) progenitors in indicated gates. (D and E) Total numbers of cells in the bone marrow (D) and spleen (E) of WT and TC10 KO animals. (F and **G**) Splenic B cells were identified using the B220 marker (B220^+^). (G) B cells were subdivided on the basis of CD21 and CD24 expression into T0-T1 cells (CD21^−^ CD24^hi^), T2-MZB cells (CD21^hi^ CD24^hi^), and follicular B cells (CD21^+^ CD24^lo^). (**H**) Flow cytometry analysis of peritoneal lavage collected from WT and TC10 KO. B cells (IgM^+^) were separated according to CD5 and CD11b into B2 (CD5^−^CD11b^−^), B1b (CD5^−^CD11b^+^), and B1a (CD5^+^CD11b^+^). Data are pooled from three independent experiments (A–C), or from one of three representative experiments with three or more mice in each group (D–F). Each dot represents an individual mouse. Student *t* test (ns: *p* > 0.05, *p < 0.05).

To specifically examine the impact of TC10 deletion on B cell development, we first analyzed the populations of B cell precursors and mature B cells in age-matched WT and TC10 KO animals. Overall bone marrow cellularity was comparable between WT and TC10 KO animals (Fig. 2D). However, we consistently observed a 20% reduction in the proportion of B cells in the bone marrow of TC10 KO mice as compared with WT controls (Fig. 2A). We further analyzed early B cell compartments according to the Hardy classification system (44), in which early B cell progenitors are identified by their high expression of CD43 and subdivided into populations A, B, and C based on BP-1 and CD24 levels (Fig. 2A, 2B). We found similar proportions of B cell progenitors A (CD24^−^BP-1^−^), B (CD24^+^BP-1^−^), and C (CD24^+^BP-1^+^) in WT and TC10 KO animals. Late CD43 negative B cell progenitors (Fig. 2A) were divided into populations D, E, and F based on IgM and IgD expression levels (Fig. 2C). Again, we did not observe any significant differences in the proportions of cells in populations D (IgM^−^IgD^−^), E (IgM^+^IgD^−^), and F (IgM^+^IgD^+^) upon TC10 deletion (Fig. 2C). Thus, although the overall amount of B cells, detected by B220 staining, was reduced in the bone marrow of TC10 KO mice, we were unable to pinpoint this reduction to any specific stage of B cell development.

Next, we analyzed B cell maturation in the peripheral lymphoid organs. Similar to the bone marrow, we again detected a slight reduction in the number of B cells in the spleens of TC10 KO mice, whereas the overall cellularity was not altered (Fig. 2E, 2F). Differential expression of CD21, CD23, and CD24 was used to distinguish follicular B cells (CD21^+^CD24^lo^), transitional T1 B cells (CD21^lo^CD24^hi^), transitional T2/marginal zone B cells (MZB) (CD21^hi^CD24^hi^, a heterogeneous population containing both T2 and MZB), and MZB cells (CD21^hi^CD23^lo^). All these populations were present in similar proportions in WT and TC10 KO animals (Fig. 2G, Supplemental Fig. 1C). We also characterized the B cell populations present in the peritoneal cavity. Cells obtained by peritoneal lavage were analyzed for IgM, CD19, CD11b, and CD5. B1a (CD5^+^CD11b^+^), B1b (CD5^−^CD11b^+^), and B2 (CD5^−^CD11b^−^) B cells were found in WT and TC10 KO in similar proportions (Fig. 2H). In contrast to the data on spleen follicular B cells, the analysis of the inguinal LNs showed normal levels of B cells (Supplemental Fig. 1D). Together, these results indicate that TC10 is nonessential for B cell maturation.

**FIGURE 3.**
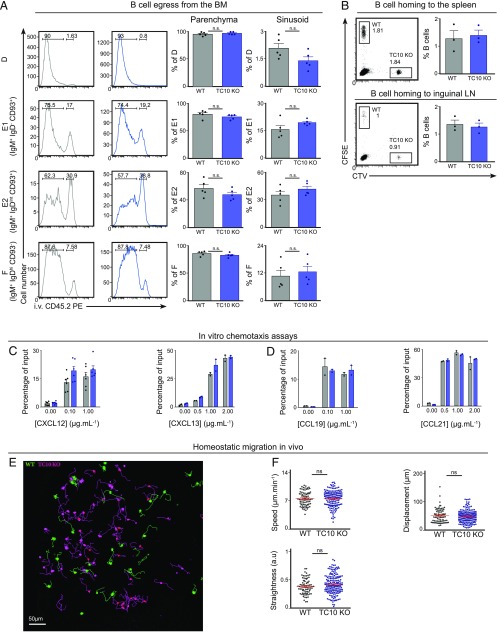
Migration, homing, and chemotactic responses are not impaired in TC10 KO B cells. (**A**) Anti-CD45 PE Ab was injected intravenously into WT and TC10 KO mice. Two minutes after injection, bone marrow was collected and stained with Abs against IgM and IgD. Populations A–D (IgM^−^IgD^−^), E1 (IgM^+^IgD^−^), E2 (IgM^+^IgD^int^), and F (IgM^+^IgD^hi^) were identified, and proportions of cells in the sinusoids (PE^+^)/parenchyma (PE^−^) were calculated on the basis of the PE labeling. Data are pooled from three independent experiments with one to two animals in each group. (**B**) CTV-labeled WT and CFSE-labeled KO naive B cells were intravenously transferred to WT recipients in a 1:1 ratio. Proportions of WT and TC10 KO B cells in secondary lymphoid organs were measured by flow cytometry 24 h after injection. (**C** and **D**) Chemotactic response of naive (C) or overnight-stimulated [with 10 μg/ml anti-IgM F(ab′)_2_] (D) WT and TC10 KO B cells to CXCL12 (C, left panel), CXCL13 (C, right panel), CCL19 (D, left panel), or CCL21 (D, right panel), at the indicated concentrations, using transwell plates. (**E** and **F**) CFSE-labeled WT and SNARF-1-labeled TC10 KO B cells were adoptively transferred to WT recipients. Then 24 h after adoptive transfer, popliteal and inguinal LN were explanted and imaged by multiphoton microscopy. Individual cells were tracked using Imaris, and average speed, mean displacement, and straightness of the tracks computed using Matlab. Quantifications of these parameters are shown in (F). Data are pooled from three independent experiments with two mice in each group (C), or representative of one of two to three independent experiments with two animals in each group (B and D–F). Student *t* test, ns: *p* > 0.05.

We then investigated if the reduced B cell numbers in the TC10 KO periphery could be explained by defective egress from the bone marrow or inefficient homing to the secondary lymphoid organs. First, to analyze the B cell egress from the bone marrow, we used an in vivo labeling method (45) where PE-labeled anti-CD45.2 Ab was intravenously injected into WT and TC10 KO animals. Two minutes after injection, animals were sacrificed and bone marrow extracted for flow cytometric analysis. In this way, we could distinguish between the B cell progenitors in the bone marrow parenchyma (PE^−^), where the Ab does not have time to enter, and those in the bone marrow sinusoids (PE^+^) readily available for the Ab staining via the blood flow. In addition to the in vivo labeling, we used labeling with IgM and IgD to distinguish between B cell progenitors at various stages of development, corresponding to Hardy fractions A–F (Fig. 2C). In particular, we subdivided fraction E in two, E1 (IgD^−^) and E2 (IgD^+^), on the basis of IgD expression. In WT mice, almost all the cells in population D were found in the bone marrow parenchyma, but 16% of the population E1 and 36% of E2 in the bone marrow sinusoids (Fig. 3A). In TC10 KO animals we observed mildly increased numbers of E1 and E2 B cells in sinusoids, potentially suggesting slower egress of mature B cells to the circulation, which could explain reduced B cell numbers in the periphery.

As Rho GTPases are major regulators of the actin cytoskeleton, which in turn plays a critical role, for example, in cell migration, we next investigated the role of TC10 in B cell migration and tissue homing. We performed adoptive transfer experiments where we injected differentially fluorescently labeled WT and TC10 KO B cells into the tail vein of the mice in a 1:1 ratio, and harvested the spleens and LNs 24 h later. Flow cytometric analysis of the tissues revealed no differences in the homing of TC10 KO B cells as compared with WT counterparts (Fig. 3B). To investigate the features of cell migration in the tissues we again adoptively transferred fluorescently labeled WT and TC10 KO B cells, and used multiphoton microscopy to detect cell migration in explanted inguinal LNs. We analyzed the individual tracks for speed, displacement, and straightness (Fig. 3E, 3F); however, TC10 KO B cells moved in a manner indistinguishable from WT B cells. Finally, to study the effects of T10 deficiency on chemotaxis in vitro, we placed isolated mouse B cells in a Boyden chamber separated from chemoattractants by a membrane with a pore size of 5 μm^2^. Using this system, we analyzed the migration of B cells toward the chemoattractants CXCL12 and CXCL13, involved in homing to the bone marrow and B cell follicles in secondary lymphoid organs, respectively. Within 3 h TC10 KO B cells transmigrated toward these chemoattractants as efficiently as WT cells, or perhaps even with slightly increased efficiency toward CXCL12 (*p* = 0.06) (Fig. 3C). We also measured the ability of TC10 KO B cells to migrate toward T cell zone chemokines CCL19 and CCL21 after BCR stimulation, but detected no differences as compared with WT cells (Fig. 3D). Together, these data indicate that TC10 is not required for B cell migration in vitro or in vivo.

**FIGURE 4.**
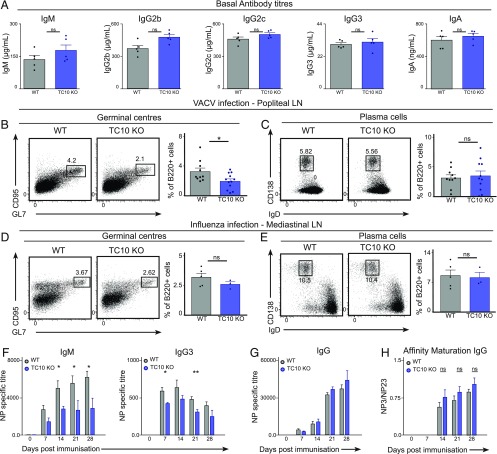
Impaired B cell responses in TC10 KO animals. (**A**) Serum was collected from naive WT and TC10 KO animals, and baseline titers of IgM, IgG2b, IgG2c, IgG3, and IgA were measured by sandwich ELISA. (**B**–**E**) WT and TC10 KO animals were infected with 10^4^ PFU VACV (B and C) or 2.10^2^ Influenza virus (D and E) by intra footpad (B and C) or intranasal injection (D and E), and the draining popliteal (B and C) or mediastinal (D and E) LNs were isolated 7 (B and C) or 9 (D and E) d later. Germinal center B cells (CD95^+^GL-7^+^ (B and D) and plasma cells [CD138^+^IgD^lo^ (C and E)] were analyzed by flow cytometry. Quantifications are shown on the panels on the right and show the percentage of B cells in the indicated gates. (**F** and **G**) WT and TC10 KO mice were immunized with NP-KLH precipitated in Alum, and serum samples collected weekly for 28 d. NP-specific IgM and IgG3 (F) titers were measured, as well as total IgG titers (G). (**H**) ELISA analysis showing affinity maturation (expressed as the ratio of NP3 to NP23 titers) of total IgG in WT and TC10 KO animals. Data are from one representative of two to three independent experiments with at least three animals in each group. Student *t* test (ns: *p* > 0.05, *p < 0.05, **p < 0.01).

An important function of B cells is to produce protective Abs. To investigate the potential role of TC10 in Ab responses, we first analyzed basal Ab levels in the serum of naive WT and TC10 KO mice by ELISA. We did not detect any statistically significant differences in the titers of IgM, IgG2b, IG2c, IgG3, or IgA in these animals (Fig. 4A). To more specifically characterize B cell responses, we infected WT and TC10 KO mice with 10,000 PFU of VACV (Western Reserve) by s.c. injections into the footpad and isolated the draining popliteal LNs 7 d later for flow cytometric analysis. To measure the effectiveness of the B cell response, we analyzed the number of germinal center B cells (identified as positive for the surface markers CD95 and GL7) in the draining LNs, formed as a response to the VACV infection. Although the initial number of LN B cells was unchanged (Supplemental Fig. 1D), interestingly, we observed a 35% decrease in the proportion of germinal center B cells in the LNs of TC10 KO animals as compared with WT animals (Fig. 4B). However, the proportions of plasma cells (IgD^lo^CD138^+^) were similar between WT and TC10 KO mice (Fig. 4C), indicating that TC10 inactivation impairs B cell engagement into the germinal center reaction after viral infection, but not the formation of extrafollicular plasma cells. To examine whether a diminished germinal center reaction was a common feature of the responses to viral infections in the absence of TC10, we next infected the mice intranasally with 200 PFU of Influenza virus (PR8 strain), and characterized the immune response in the mediastinal LNs 9 d postinfection. Although we observed a trend of reduction in the germinal center response in TC10 KO mice compared with WT, this was not statistically significant in our experimental cohort (Fig. 4D). Again, we found no indications of defected plasma cell generation (Fig. 4E).

The triggering and success of the germinal center reaction strongly depends on the appearance of follicular helper T cells (Tfh). To test whether the defect presented by TC10 KO animals could result from impaired Tfh formation, we first analyzed the Tfh numbers in popliteal and mediastinal LNs upon VACV and influenza infections, respectively, and found them undiminished in TC10 KO mice (Supplemental Fig. 2A, 2B). To determine if the effect on germinal center reaction was B cell intrinsic or if the functionality of Tfh might also be affected by the systemic gene deletion, we generated bone marrow chimeric mice. We lethally irradiated μMT mice, lacking B cells, and reconstituted the hematopoietic compartment by bone marrow containing 80% from μMT and 20% from WT or TC10 KO background. We used this model for VACV infections and measured the numbers of germinal center B cells and plasma cells (Supplemental Fig. 2C, 2D). Notably, we again saw a reduced number of TC10 KO germinal center B cells as compared with WT (30% reduction, *p* value 0.0646), but similar numbers of plasma cells. Also, the numbers of Tfh remained normal (Supplemental Fig. 2E). Together, these data suggest that TC10 plays a B cell intrinsic role in the germinal center reaction upon viral infections.

The observed defects in the induction of germinal center B cells prompted us to study T cell–dependent immune responses in more detail using the model system of NP-conjugated KLH. We immunized TC10 KO and age-matched WT mice with i.p. injection of NP-KLH precipitated in Alum and collected serum samples weekly for 28 d. The levels of NP-specific Abs were measured by ELISA. We observed a consistent 50% reduction in IgM titers, as well as a 25% reduction in the IgG3 titers in TC10 KO mice as compared with WT controls (Fig. 4F). In contrast, WT and TC10 KO mice exhibited a comparable increase in total IgG titers over the course of the experiment (Fig. 4G). We next asked whether the IgG Abs produced in TC10 KO animals had undergone affinity maturation by comparing binding of IgG Abs to NP23-BSA, measuring both lower and higher affinity Abs, and NP3-BSA, which preferentially detects the higher affinity Abs. By this readout for affinity maturation, we detected similar affinity maturation in both WT and TC10 KO mice (Fig. 4H). Together, these results suggest that TC10 plays a role in the early steps of the immune response, as indicated by defective IgM and IgG3 responses. However, this deficiency is overcome by sufficient generation and affinity maturation of other Ab isotypes in later stages of the immune response.

**FIGURE 5.**
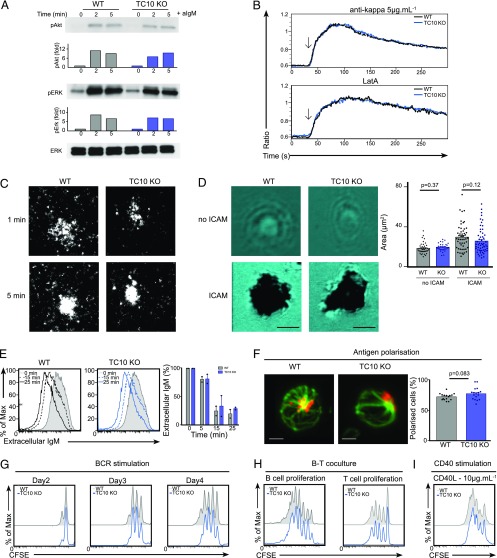
BCR signaling is not impaired in TC10 KO B cells. (**A**) B cells isolated from WT and TC10 KO mice were stimulated with 2 μg/ml anti-IgM F(ab′)_2_ for 0, 2, or 5 min, and phosphorylation of Akt (S473) and ERK were detected by Western blotting with phosphospecific Abs. Graphs below each lane indicate the relative intensity of each band, normalized to the level of total ERK, relative to pAkt or pERK at t0. (**B**) Intracellular calcium influx in purified, Indo-1 labeled WT or TC10 KO B cells after stimulation with 5 μg/ml anti-κ or 1 μM Latrunculin A. (**C**) WT and TC10-deficient B cells were settled on labeled Ag-loaded planar lipid bilayers and imaged with a TIRF microscope. Distribution of Ag was analyzed over time, and representative images of the cells after 1 and 5 min are shown. (**D**) WT and TC10 KO B cells were settled on planar lipid bilayers loaded with limiting amounts of Ag, with or without ICAM, and imaged by interference resonance microscopy. Areas of tight contact (appearing in black on the images) between the cell and the bilayers were measured using Image J and quantifications are shown on the right. (**E**) Purified naive WT or TC10 KO B cells were labeled on ice with soluble biotinylated anti-IgM, and incubated at 37°C for the indicated times, prior to fixation. Extracellular Ag was then detected by flow cytometry. Data are quantified on the right panel as percentage of initial mean fluorescence intensity for each sample. (**F**) Purified naive WT or TC10 KO B cells were labeled on ice with AlexaFluor 647 tagged anti-IgM F(ab′)_2_ and incubated for 30 min at 37°C prior to fixation, stained with an anti-tubulin Ab (green), and imaged by confocal microscopy. The fraction of cells exhibiting polarized Ag (red) distribution on each image was measured, and quantification is shown on the right. (**G**) Purified CFSE-labeled WT or TC10 KO B cells were cultured with 10 μg/ml anti-IgM F(ab′)_2_ in presence of IL4 and proliferation was monitored by flow cytometry at day 1, 2, and 3 after stimulation. (**H** and **I**) Purified CFSE-labeled WT or TC10 KO B cells were stimulated with beads coated with IgM and OVA and cocultured with CTV-labeled OTII T cells (H) or cultured with 10 μg/ml CD40L in presence of IL4 (I), and proliferation was monitored by flow cytometry at day 3 after stimulation. Data are representative of two to three independent experiments. Student *t* test, ns: *p* > 0.05.

As we observed defects in the early B cell responses to immunization in vivo, we next sought to investigate the early B cell activation in more detail by analyzing the responses of the isolated primary B cells to the activation of the BCR in vitro. For this, we studied the activation of the downstream kinases ERK1/2 and Akt by measuring phosphorylation of these proteins after BCR engagement using immunoblotting. We detected a 35% reduction of the early Akt phosphorylation and delayed kinetics of this pathway (Fig. 5A). In addition, we observed slightly reduced ERK phosphorylation, indicating mild defects in the capability of the BCR to trigger this downstream pathway (Fig. 5A). In contrast, analysis of pSrc, pSHP1, and pPLCγ2 did not reveal alterations as investigated during the first 10 min after activation (data not shown). We also used a ratiometric calcium binding dye Indo-1 in flow cytometry to measure calcium mobilization upon BCR activation either by anti-BCR stimulation or the disruption of the actin cytoskeleton, which has been shown to trigger BCR signaling (8–10). There were no detectable differences in calcium signaling in TC10 KO B cells as compared with WT cells (Fig. 5B). Next, to mimic B cell activation by APCs, we used planar lipid bilayers tethered with anti-BCR as surrogate Ag and visualized the B cell spreading response and Ag gathering in high definition using TIRF microscopy, or interference resonance microscopy. B cells from TC10 KO mice spread and gathered Ag microclusters in a similar manner to WT cells as visualized by TIRF microscopy (Fig. 5C). To test cell spreading on bilayers with lower Ag avidity, we used interference resonance microscopy either with or without ICAM, but did not detect differences between WT and TC10 KO cells (Fig. 5D).

An inherent part of B cell activation is BCR-mediated Ag internalization for further intracellular processing and presentation to T cells. As TC10 has been indicated in vesicle traffic and exocytosis in other systems, we first set out to determine the intracellular localization of TC10. Although we did not get any of the available anti-TC10 Abs working in immunofluorescence, we used TC10 linked to mCherry fluorescent protein. We found that in A20 lymphoma B cells, TC10 localized both at the plasma membrane, in a mostly homogenous manner, as well as intracellular vesicles. By coexpression of TC10-mCherry and various GFP-fused Rab-proteins or labeling the lysosomal compartment with lysotracker, we observed a partial colocalization with lysotracker and the late endosomal marker Rab7 (Supplemental Fig. 3A, 3C). TC10 did not colocalize with the early endosomal marker Rab5 (Supplemental Fig. 3B). Based on this microscopic data, TC10 might play a role in vesicular traffic in B cells. However, we did not detect TC10 to colocalize with internalized Ag (Supplemental Fig. 3D) or change its localization pattern upon Ag stimulation (Supplemental Fig. 3D). Moreover, when A20 cells were settled on planar lipid bilayers, TC10 was not recruited to Ag microclusters or to the plasma membrane (Supplemental Fig. 3E). To investigate the role in vesicle traffic using functional assays, we first set out to measure BCR internalization by detecting surface-retained biotinylated anti-BCR at 0, 15, and 25 min, but found no distinguishable differences between WT and TC10 KO B cells (Fig. 5E). We then looked at the fate of Ag after internalization by analyzing the polarization of Ag in one pole of the cell, which was also identical in WT and TC10 KO cells (Fig. 5F). To look at the B cell proliferation triggered by BCR signaling, we labeled the cells with CFSE and followed its dilution for 3 d; however, we could not detect any significant differences between WT and TC10 KO B cells (Fig. 5G, Supplemental Fig. 4A). Survival rates of WT and TC10 KO B cells under such conditions were similar (data not shown). Furthermore, as vesicle traffic is required for Ag processing and MHC class II–mediated Ag presentation to T cells, we investigated if the B cell to T cell conjugation might be hampered in the absence of TC10 KO. For this, we followed the proliferation of both B and T cells in a coculture (Fig. 5H, Supplemental Fig. 4C) indicative of the amount of activating signals the cells receive from each other. We found no defects caused by a lack of TC10. Similarly, we also found that TC10-deficient B cells were able to respond normally to CD40L, a major activating signal received from T helper cells. (Fig. 5I, Supplemental Fig. 4B). These data suggest that TC10 KO B cells are fully proficient in sensing and responding to T cell help. Based on this set of data, we concluded that TC10-deficient B cells can overcome the lowered activation of ERK1/2 and Akt and show no significant defects in activation triggered by BCR agonists in vitro.

**FIGURE 6.**
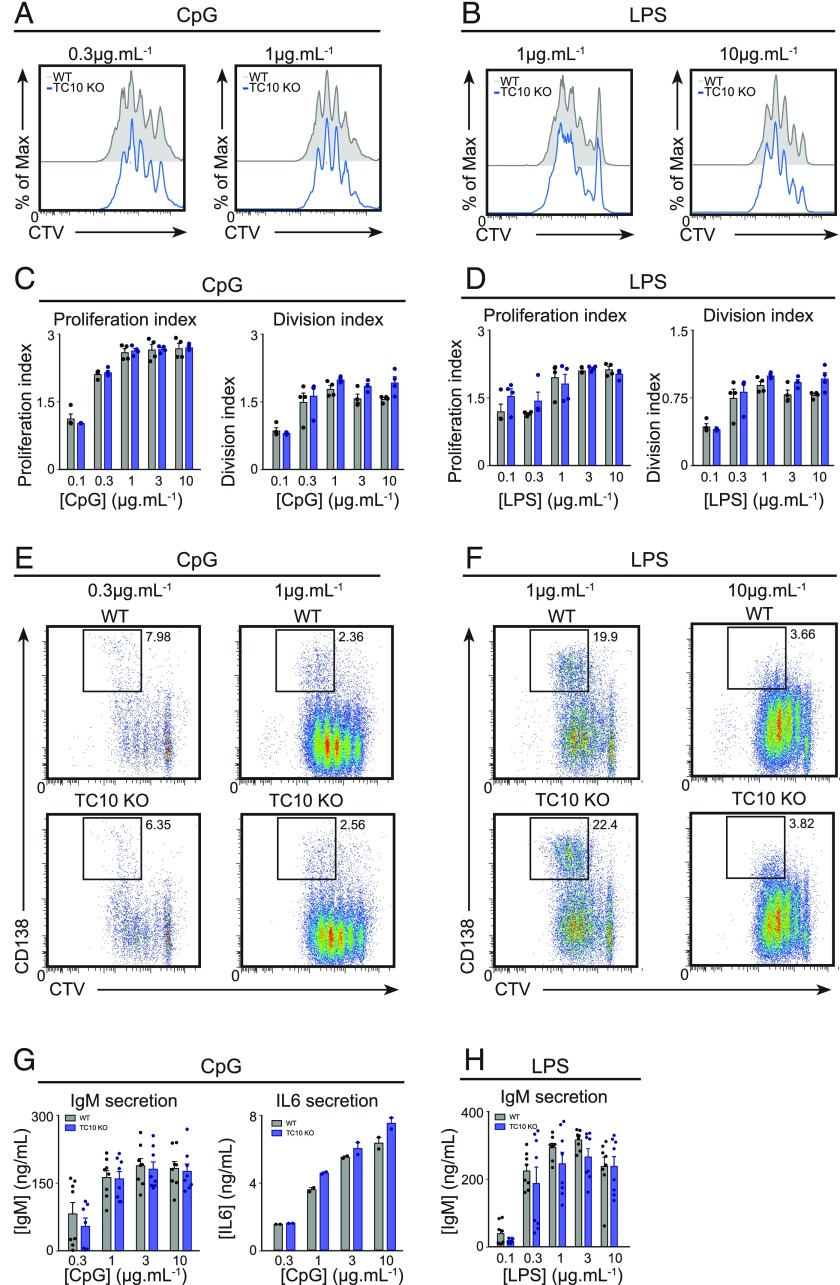
B cell activation by TLR ligands is not impaired in B cells from TC10 KO mice. CTV-labeled naive B cells isolated from WT and TC10 KO animals were stimulated with CpG (**A**, **C**, **E**, and **G**) or LPS (**B**, **D**, **F**, and **H**) at the indicated concentrations. Proliferation [CTV dilution peak (A and B) and proliferation/division indices (C and D)] and plasma cell differentiation [CD138^+^ (E and F)] were measured after 3 d by flow cytometry. Also after 3 d, secreted IgM (G and H) or IL-6 (G) in the supernatant was measured by ELISA. Data are representative of three independent experiments.

B cells can be efficiently stimulated by the engagement of TLR 4 and 9 ligands LPS and CpG DNA, respectively. We measured responses to two different concentrations of LPS and CpG in WT and TC10 KO mouse primary B cells by analyzing cell proliferation using CTV dilutions (Fig. 6A–D) and plasma cell differentiation marked by expression of CD138 (Fig. 6E, 6F). TC10 KO primary B cells behaved similarly to WT B cells in these assays, indicating that TC10 is not required for proliferation and differentiation downstream of TLR 4 and 9. To investigate if TC10 could play a general role in exocytosis in B cells, we measured secretion of IgM and IL-6 upon stimulation with CpG and the secretion of IgM upon stimulation with LPS (Fig. 6G, 6H). We did not detect any diminution in the secretion following CpG stimulation. The ability of TC10 KO B cells to secrete IgM upon LPS stimulation also appeared normal, although with increased variability in efficiency. Overall, we conclude that TC10 KO B cells show no significant defects in the responses to TLR signals.

**FIGURE 7.**
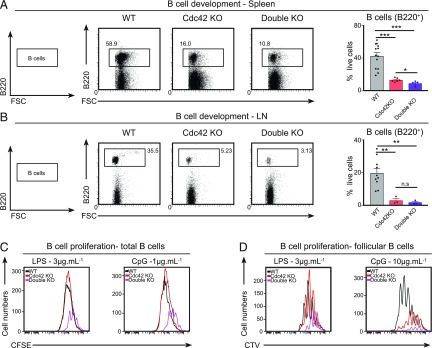
TC10 and Cdc42 cooperate in proliferative responses to TLR agonists. (**A** and **B**) Flow cytometry analysis of spleen (A) and inguinal LNs (B) of WT, Cdc42 KO, and CD42 KO TC10 KO mice. B cells were identified as B220^+^. Data are pooled from three to four independent experiments with one to two mice in each group. The data on B cells in LN of Cdc42 KO animals were published in a previous study (22), without the information on double KO animals. (**C** and **D**) CTV-labeled naive total (C) or sorted follicular (D) WT, Cdc42 KO, or double KO B cells were stimulated with LPS (3 μg ml^−1^) or CpG (10 μg ml^−1^) and proliferation was measured after 3 d. Data are representative of three independent experiments. Student *t* test (*p < 0.05, **p < 0.01, ***p < 0.001). n.s, *p* > 0.05.

As TC10 belongs to the same subfamily of Rho GTPases as Cdc42, and Cdc42 is critical for various aspects of B cell maturation, activation, and differentiation into plasma cells (22), we sought to examine the phenotype of B cells deficient in both TC10 and Cdc42. Double KO mice were generated by crossing TC10 KO mice with a conditional mouse model where Cdc42 is deleted only in B cells (22). Despite the strong effect of Cdc42 alone in B cell development, we detected a significant additional reduction from 14 to 8.5% in total splenic B cell numbers in the TC10/Cdc42 double KO mice (Fig. 7A), further supporting the reduction seen in the TC10 KO single strain (Fig. 2A). In contrast, but in line with what we observed in TC10 KO animals, B cells were found in similar proportions in LNs from Cdc42 KO and TC10/Cdc42 double KO mice (Fig. 7B). Interestingly, when looking at the B cell proliferation upon TLR signaling, double KO cells showed a clear defect in proliferation, whereas Cdc42 KO alone proliferated normally (Fig. 7C). When performing the same experiment with sorted follicular B cells, we observed a defect in double KO cells upon LPS stimulation, whereas Cdc42 KO cells proliferated normally. Upon CpG stimulation, Cdc42 KO cells were also defected, which, however, was more severe in double KO cells (Fig. 7D). Together, these experiments showed that although TC10 and Cdc42 seem to have an additive function during B cell maturation, they do appear to co-operate in TLR-induced proliferation, such that the defects observed mostly required the deletion of both proteins.

## Discussion

The critical role of the Rho family GTPases in lymphocyte activation has become evident; however, not much is known about the functions of the Rho family members outside of the highly conserved triplet of Cdc42, Rac1/2, and RhoA. In this study, we focused on TC10, a poorly studied Rho GTPase that bears close homology to Cdc42, a critical regulator of B cell function. To study the physiological role of TC10 and assess its function upon immunization or infection, we generated a TC10-deficient mouse, which, to the best of our knowledge, is the first KO mouse model generated for TC10. Our studies demonstrate that TC10 has an immunomodulatory role in the germinal center reaction triggered by viral infections and is required for effective IgM responses upon T cell–dependent immunization. By generating a TC10/Cdc42 double KO strain, we show that both proteins additively affect B cell maturation and synergistically regulate B cell proliferation in response to TLR signaling.

The proportionally normal B cell populations in TC10 KO mice indicated that there is no specific block or inhibition at any given transition during B cell development and maturation. Nevertheless, we detected a reduction in the absolute numbers of both immature and mature B cells in the bone marrow and in the spleen, respectively (Fig. 2A, 2F). Although the overall cellularity was not altered, this reduction in B cell numbers suggests a specific regulatory role for TC10 in cell survival or in the development of the first B cell progenitors from the common lymphoid progenitors. Despite a dramatic reduction in B cell numbers observed in Cdc42-KO mice ((22); Fig. 7A), we detected an even greater reduction in TC10/Cdc42 double KO mice, indicating nonredundant roles of TC10 and Cdc42 in B cell development (Fig. 7A).

Our studies were sparked by the recently published critical role of Cdc42 in the germinal center reactions and the generation of plasma cells (22); however, we did not detect defects of comparable severity in our TC10 KO mouse model. Nevertheless, a 50% reduction in the production of IgM and 25% reduced IgG3 were detected upon immunization, indicative of defects in the early humoral response (Fig. 4F). Upon VACV infection, TC10-deficient B cells formed fewer germinal center B cells as compared with their WT counterparts (Fig. 4B), a result that was supported by a similar finding using another viral infection model, Influenza virus (Fig. 4D). The formation of plasma cells remained unaffected, suggesting compensation from the nongerminal center–derived, extrafollicular plasma cells. Cdc42 is also required for efficient B cell homeostatic migration in the LNs in vivo (22). On the contrary, we found that TC10-deficient B cells did not show any alteration in their migration or were even slightly faster in migration than WT cells (Fig. 3C–F). We examined early BCR signaling and found normal calcium flux responses in the TC10-deficient B cells, whereas Akt and ERK phosphorylation were slightly diminished and trailing. However, the cells seemed to be able to overcome this and did not show detectable defects in the formation of the immunological synapse (Fig. 5). These data suggest that TC10 is largely redundant for the processes that are regulated by Cdc42. Interestingly, however, generation of a TC10/Cdc42 double KO strain revealed that TC10 and Cdc42 function together in cell proliferation in response to TLR signaling (Fig. 7C, 7D). TC10 was recently identified as a critical gene in the gene regulatory network analysis in canine lymphoma, suggesting a potential role in B cell activation status (46). This would be indicative of a fine-tuned role of TC10 in B cell activation, which our data would support, that could predispose the cells to lymphomagenesis.

The defective Ab and germinal center responses in TC10 KO mice we found in this study appear rather specific in their nature. In our study, the experiments were performed with mice that lacked TC10 in all tissues. To address the question of whether the defect in the germinal center responses was B cell intrinsic, we generated bone marrow chimeras, where specifically the B cell compartment originated from TC10 KO marrow. In this system, TC10 KO B cells appeared, again, less able to form germinal center B cells upon VACV infection (Supplemental Fig. 2), indicating that the phenotype is B cell intrinsic. The underlying molecular mechanisms remain to be further dissected in future studies. The mechanistic dissection of TC10 function would strongly benefit from Abs that recognize TC10 in fixed tissues and cell samples. We were not able to obtain reliable immunofluorescent staining for TC10 by using commercially available Abs or Abs raised by ourselves (data not shown). As an alternative, we visualized the localization of TC10-mCherry fusion protein and found localization to the plasma membrane as well as to intracellular vesicles (Supplemental Fig. 3), suggesting a role in vesicle traffic, in line with the literature in other cell types. In adipocytes, TC10 is critical for insulin-triggered GLUT4 translocation to the cell membrane. Mechanistically, this appears to involve triggered localization of TC10 to the plasma membrane, where TC10 functions as a target for Exo70 and exocytic vesicle docking (26, 47, 48). In neurons, TC10 promotes neurite elongation again by promoting Exo70 recruitment and exocytosis (27). These functions in exocytosis occur downstream of particular cellular signals. In B cells, the counterparts for the cellular events regulated by TC10 in adipocytes or neurons are not obvious. If TC10 was critical for exocytosis in B cells, one might expect to detect general defects in the surface expression of BCR and peptide-MHC class II molecules, or in Ab secretion. Reduced IgM and IgG3 secretion in TC10 KO mice upon protein Ag immunization could reflect a specific defect in exocytosis, but might also be indicative of multiple other cellular events linked to class-switching. As we found other Ab isotypes to be secreted in sufficient amounts, as well as IgM and IL-6 to be secreted normally upon TLR stimulation, it is unlikely that TC10 has a major role in exocytosis in B cells, or the effect in exocytosis is limited to specific conditions. Furthermore, the other detected phenotypes, B cell maturation and the germinal center reaction, do not propose clear links to vesicular pathways. It is also important to note that the reported studies on TC10 in adipocytes and neurons have not been carried out in TC10-deficient animal models, so there is a significant lack of data obtained using physiological model systems.

Taken together, by generating a TC10-deficient mouse model and analyzing its immunological phenotype, we showed that TC10 is not required for normal B cell development and maturation. However, we detected defective IgM and IgG3 responses upon T cell–dependent immunizations and diminished numbers of germinal center B cells upon VACV infection in TC10 KO mice, indicative of immunomodulatory functions of TC10.

## Supplementary Material

Data Supplement
