# Supplementary material for: The Small Rho GTPase TC10 Modulates B Cell Immune Responses
Source: J Immunol. 2017 Jul 26;199(5):1682–95. doi: 10.4049/jimmunol.1602167 (PMC5563166; doi:10.4049/jimmunol.1602167)
Supplement: Data Supplement [file JI_1602167.zip › JI_1602167_Supplemental_Figures_1.pdf]

Figure S1

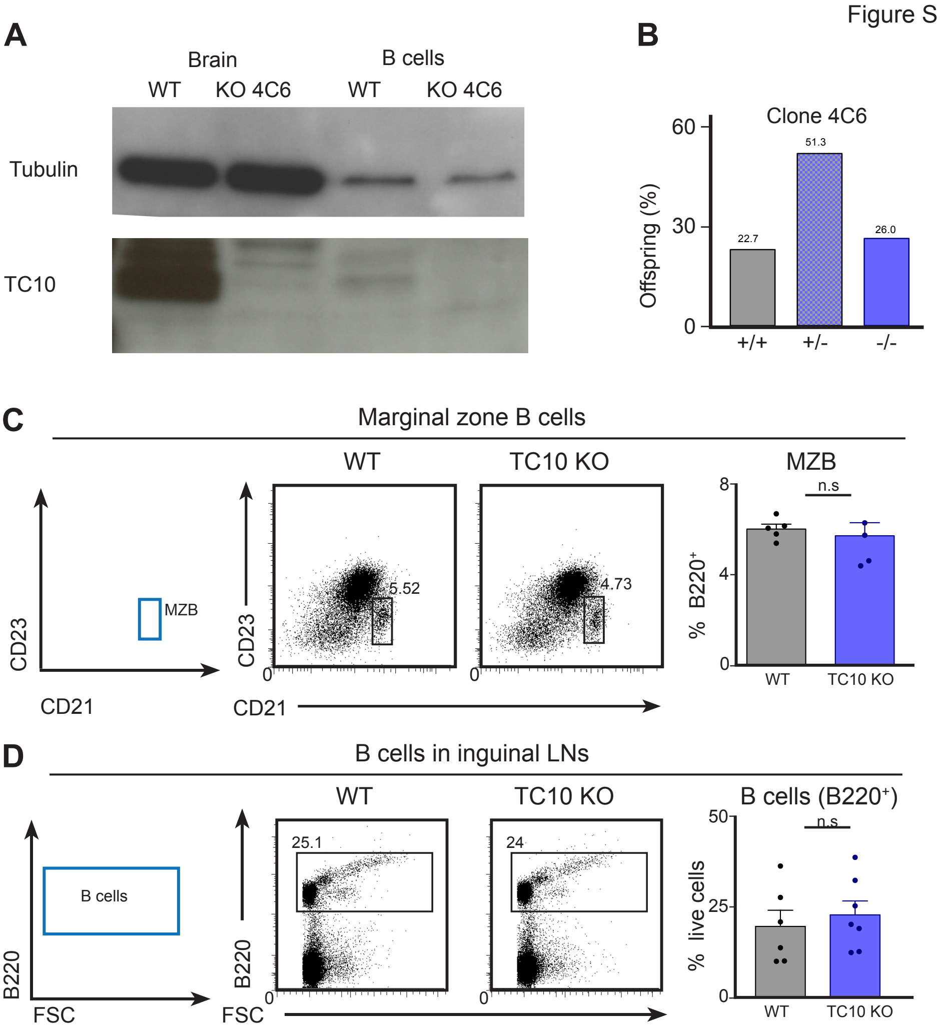

Figure S1 (Related to Figure 1 and 2): TC10 KO mice show normal marginal zone B cell subpopulations and B cells in inguinal LNs.

A) Expression of TC10 (lower panel) and α-tubulin (upper panel) were detected by Western Blot in total protein extract from brain or purified B cells of WT and TC10-deficient animals originating from the ES cell clone 4C6. B) Transmission frequencies (calculated over 100-200 pups from heterozygous breedings) of the TC10 KO allele in animals originating from the ES cell clone 4C6. C) Marginal zone B cells ($CD21^{hi}$ $CD23^{-}$) were identified from the total B cell ($B220^{+}$) population. D) B cells in inguinal LNs from WT and TC10 KO animals were identified as $B220^{+}$. Data were pooled from 2 (C) or 3 (D) independent experiments with 2-3 mice in each group

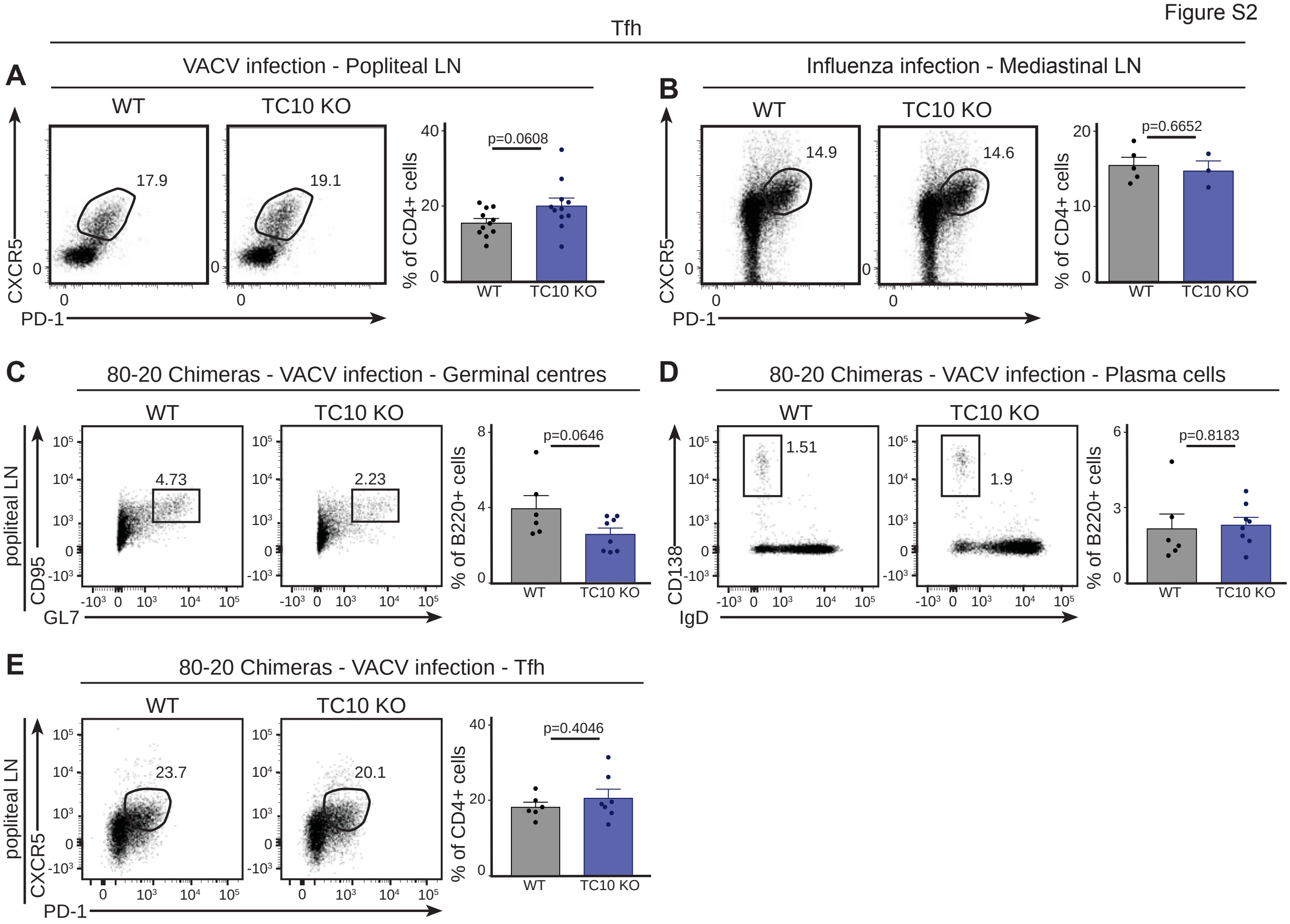

Figure S2 (Related to figure 4): B cell intrinsic role of TC10 in germinal center reactions.
A-B) WT and TC10 KO animals were infected with 104 PFU Vaccinia virus (A) or 2.10^2 Influenza Virus (B) by intra-footpad (A) or intranasal injection (B), and the draining popliteal (A) or mediastinal (B) lymph nodes were isolated 7 (A) or 9 (B) days later. Tfh cells (CXCR5$^+$PD-1$^+$) were analysed by flow cytometry. C-E) Lethally irradiated μMT mice were reconstituted for 8 weeks with 80% μMT BM and 20% of WT or TC10 KO BM, infected with 104 PFU Vaccinia virus, and popliteal LN analysed by flow cytometry 7 days later. GC B cells (CD95$^+$GL-7$^+$, C), plasma cells (CD138$^+$IgD^{lo}, D) and Tfh cells (CXCR5$^+$PD-1$^+$) were analyzed. Data are representative of 2-3 (A-D) or 1 experiments. Each dot represents an individual mouse. Student's t-test (ns: p>0.05, *: p<0.05, **: p<0.01).

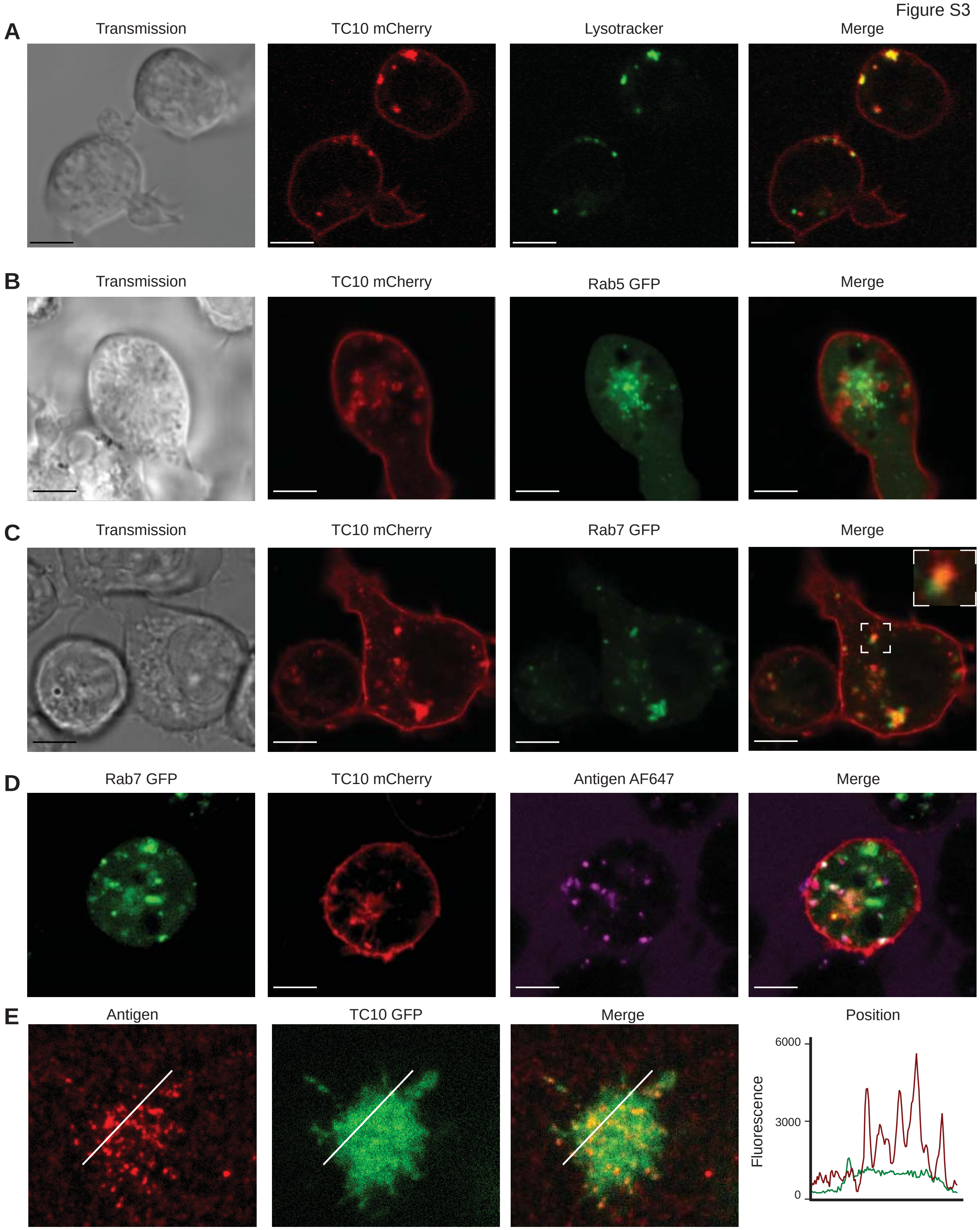

Figure S3: TC10 associates with the plasma membrane and with late endosomal vesicles, but is not recruited to antigen-containing structures.

A-D) A20 lymphoma cells expressing a HEL-specific IgM BCR were transfected with TC10-mCherry and Rab5-GFP (B) or Rab7-GFP (C) and when specified stained with lysotracker (A), settled on fibronectin-coated slides, and imaged by confocal microscopy. One representative slice is shown. (D) A20 cells settled on fibronectin-coated slides were stimulated with AlexaFluor-647 anti-IgM antibodies, and imaged by confocal microscopy after 15 minutes. One representative slice is shown. (E) A20 lymphoma cells expressing a HEL-specific IgM BCR were transfected with TC10GFP, settled on HEL-loaded lipid bilayers, and imaged by TIRF microscopy. Signal intensities of antigen (in red) and TC10GFP (in green) along the white line are plotted on the right. Scale bars – 3μm.

Figure S4

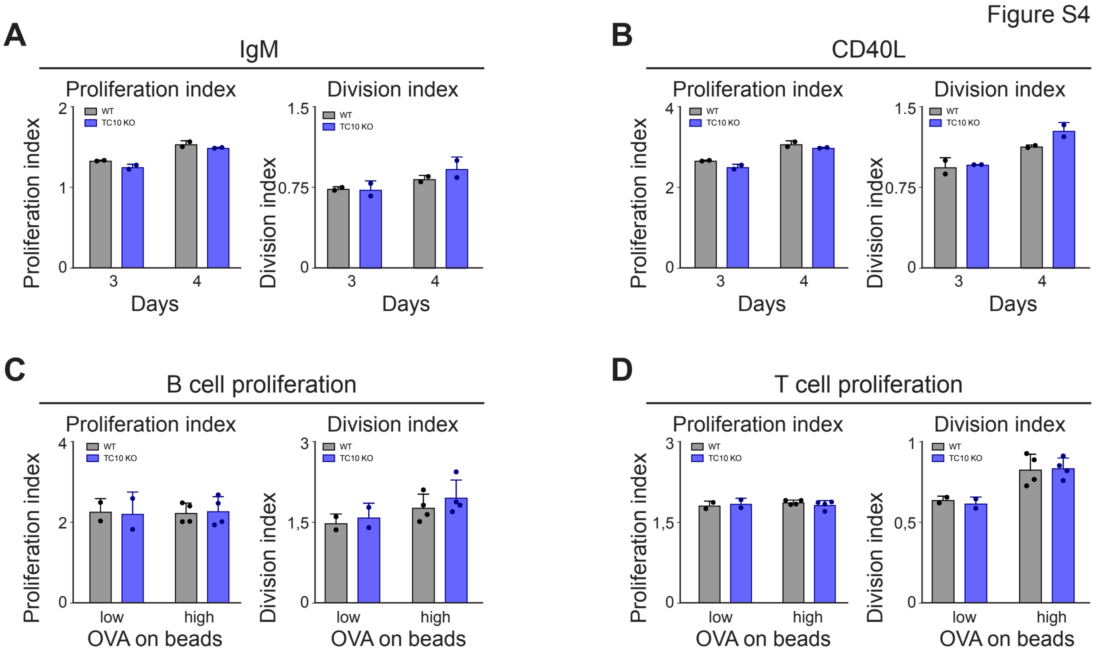

Figure S4 (Related to figure 5): Similar proliferation profiles of WT and TC10 KO B cells.
A-B) Naïve CFSE-labelled WT or TC10 KO B cells were stimulated with anti-IgM (A) or CD40L (B) in presence of IL4. Proliferation and division indices were calculated using Flowjo at day 3 and day 4. C-D) Naïve CFSE-labelled WT or TC10 Ko B cells were stimulated with beads coated with anti-IgM and OVA and cocultured for 3 days with OTII T cells. Proliferation and division indices for B (C) and T (D) cells were calculated at day 3 using Flowjo. Data are representative of 3 independent experiments with at least 2 mice in each group.
